# Genetic Engineering of *Escherichia coli* BL21 (DE3) with a codon-optimized insecticidal toxin complex gene *tccZ*

**DOI:** 10.1099/acmi.0.000426

**Published:** 2023-01-24

**Authors:** Mosibudi Thabiki Malomane, Kulsum Kondiah, Mahloro Hope Serepa-Dlamini

**Affiliations:** ^1^​ Department of Biotechnology and Food Technology, Faculty of Science, University of Johannesburg, Doornfontein Campus, PO Box 17011, Doornfontein 2028, Johannesburg, South Africa

**Keywords:** *Escherichia coli* BL21(DE3), insecticidal toxin, pET SUMO, *Pantoea ananatis* strain MHSD5, *tccZ* gene, toxin complex

## Abstract

A toxin complex consists of a high-molecular-weight group of toxins that exhibits insecticidal activity against insect pests. These toxins are a promising alternative to *Bacillus thuringiensis* (*Bt*) toxins that have been extensively utilized in insect pest control. Herein, a codon-optimized insecticidal gene (*tccZ*) (381 bp) identified in *Pantoea ananatis* strain MHSD5 (a bacterial endophyte previously isolated from *Pellaea calomelanos*) was ligated into the pET SUMO expression vector and expressed in *Escherichia coli* BL21 (DE3). We report the success of cloning the *tccZ* gene into the pET SUMO vector and ultimately the transformation into *E. coli* BL21 (DE3) competent cells. However, despite conducting a time course of expression as well as isopropyl β-d-1-thiogalactopyranoside (IPTG) dosage optimization to determine optimal conditions for expression, TccZ protein expression could not be detected on Stain-Free and Coomassie-stained SDS-PAGE gels.

## Full-Text

## Data Summary

All data required for the reproduction of this work have been provided here. This work formed part of a master’s degree study at the University of Johannesburg, which can be found at https://hdl.handle.net/10210/498390

## Introduction

*Pantoea ananatis* strain MHSD5 is an endophytic bacterium that was isolated from surface-sterilized leaves of a South African medicinal plant, *Pellaea calomelanos* [1]. *P. calomelanos*, commonly known as hard fern, is a medicinal plant that belongs to the family *Pteridaceae*, and its burnt leaves produce smoke that is used for treatment of chest ailments [2]. Whole-genome sequencing of *P. ananatis* strain MHSD5 revealed the presence of the *tccZ* insecticidal gene, which forms part of a toxin complex (Tc) comprising high-molecular-weight insecticidal toxins with oral and injectable toxicity towards insects [3, 4]. The Tc comprises three components (A, B and C), all of which play a role in insecticidal activity [5–7]. Genes belonging to the toxin complex were first characterized in entomopathogenic nematode-associated bacteria [8, 9]. The *tccZ* gene has been reported in an Australian and US isolate of *Photorhabdus asymbiotica* [10]. Whilst the TcA–TcB–TcC complex is responsible for insecticidal activity, TccZ has no known function. The *tccZ* gene has been reported to occur at the end of the operons encoding Tcs and is tightly linked to the *tcc*–toxin loci [10]. In addition, the toxin complexes have been reported in genomes of bacterial species that are not associated with insects and entomopathogenic nematodes [11]. These toxins are reported to be involved in pest control of various insect hosts [12–14].

Previously, *Bacillus thuringiensis* (*Bt*) have been used for biopesticides formulations, as well as transgenic crop deployment, and they have proven to be effective in controlling insect pests [15]. However, many insects have adapted and developed resistance against *Bt* toxins [15–17]. Consequently, this makes toxin complex genes such as *tccZ* an important contributor in the agricultural industry, as well as promising alternatives to the already extensively utilized *Bt* toxins [4, 18, 19].

The objective of this study was to transform *Escherichia coli* BL21 (DE3) with a synthetic *tccZ* gene ligated to the pET SUMO expression vector and to further express the recombinant TccZ protein at optimal concentrations. The sequence of the *tccZ* gene from *P. ananatis* strain MHSD5 was codon-optimized, synthesized and used for the purpose of protein expression in *E. coli *. Codon optimization allows for better expression of non-native genes in *E. coli *.

## Methods

### Codon optimization and *tccZ* gene synthesis

The *tccZ* gene sequence obtained from the genome of *P. ananatis* strain MHSD5 (accession number PUEK01000000) was used in this study. To facilitate high expression of TccZ protein in *E. coli* BL21 (DE3), the gene sequence was codon-optimized using a manual codon harmonizer tool [20]. The harmonized gene was sent to Inqaba Biotechnical Industries (Pty) Ltd, Pretoria, South Africa, for synthesis and ligation into pET SUMO vector.

### Transformation of One Shot Mach-1-T1 *E. coli*

Two microlitres of pET SUMO/*tccZ* (pET SUMO ligated with *tccZ* gene) and pUC19 plasmid DNA (used as positive plasmid control) were used to transform 50 µl aliquots of One Shot Mach1-T1 chemically competent *E. coli* cells via the heat shock method as described here. The vials were incubated on ice for 30 min, followed by heat shock for 30 s at 42 °C in a non-shaking water bath. The vials were immediately transferred to ice and 250 µl Super Optimal broth with catabolite repression medium (S.O.C) was added to each vial, and these were then placed horizontally in a shaking incubator (200 r.p.m.) at 37 °C for 1 h. To ensure that clear and evenly distributed colonies were obtained, two volumes (100 and 150 µl) from each transformation were spread on a prewarmed Luria–Bertani (LB) agar plate containing 50 µg ml^−1^ kanamycin and incubated overnight at 37 °C. The plates were observed for colony growth.

The transformation efficiency (c.f.u. µg^−1^) was calculated as follows:

$$\frac{Number\;of\;transformats\;(c.f.u)}{DNA\;added\;to\;the\;cells\;(\mu g)}\times \frac{Volume\;of\;transformation\;(\mu L)}{Volume\;of\;cells\;plated\;(\mu L)}\times Dilution\;factor$$

where:

DNA added to the cells=0.01 µg

olume of cells plated=100 µl

volume of transformation=300 µl

### Colony polymerase chain reaction

Primers specific to the *tccZ* gene were designed manually using OligoAnalyzer Tool (Integrated DNA Technologies, idtdna.com). The forward and reverse sequence were 5′-ATGCTTAAATCCGCGCTTTTTCTC-3′ and 5′-CTAATTACGCTTAACCCCATAGTC-3′, respectively. To confirm successful transformation, half a colony was mixed with 12.5 µl 2× Master Mix, 10.5 µl of nuclease-free water and 1 µl of each primer (10 µM) to make up a total of 25 µl reaction for PCR. Amplification of the *tccZ* gene was carried out in a Bio-Rad T100 Thermocycler (Bio-Rad, USA) using the following parameters: initial denaturation at 94 °C for 30 s, followed by 35 cycles at 94 °C for 30 s, 50 °C for 30 s and 68 °C for 1 min, and a final extension at 68 °C for 10 min. The PCR amplicons were confirmed by electrophoresis on 1 % agarose gel (90 V for 40 min) with 50 bp DNA ladder (New England Biolabs, USA). The other half of the colony was spread on LB agar supplemented with 50 µg ml^−1^ of kanamycin and incubated overnight at 37 °C (to be used for plasmid extraction).

### Plasmid extraction and sequencing

Ten single colonies were selected from overnight LB–kanamycin agar plates and each colony was inoculated into 5 ml LB broth supplemented with 50 µg ml^−1^ kanamycin. The cultures were incubated in a shaking incubator (200 r.p.m.) and grown overnight at 37 °C. Following incubation, 3 ml of each culture was centrifuged (Thermo Scientific, UK) at 11 000 ***g*** for 1 min and the supernatants were discarded.

Plasmid DNA (One Shot Mach-1-T1 *E. coli/*pET SUMO/*tccZ*) was extracted and column-purified using the ZR Plasmid DNA Miniprep Classic kit (Zymo Research, USA) following the manufacturer’s protocol. The extracted plasmid DNA was sent for sequencing at Inqaba Biotechnical Industries (Pty) Ltd, Pretoria, South Africa. The pET SUMO forward sequencing primer (5′-AGATTCTTGTACGACGGTATTAG-3′) and T7 reverse sequencing primer (5′-TAGTTATTGCTCAGCGGTGG-3′) were used for sequencing.

Positive transformants carrying the full-length *tccZ* gene in the correct orientation were propagated in LB media containing 50 µg ml^−1^ kanamycin and maintained as glycerol stocks for downstream applications.

### Transformation of One Shot *E. coli* BL21 (DE3) for protein expression

The One Shot *E. coli* BL21 (DE3) cells are specifically designed to express genes that are regulated by a T7 promoter [19]. Following the manufacturer’s protocol, plasmid DNA from a positive transformant (One Shot Mach-1-T1 *E. coli */pET SUMO/*tccZ*) was extracted using the NucleoSpin Microbial DNA kit (Macherey-Nagel, Germany). The pET SUMO/*tccZ* and pET SUMO-CAT (used as positive expression control) were transformed into One Shot *E. coli* BL21 (DE3) as described previously. Ten millilitres of LB broth containing 50 µg ml^−1^ kanamycin and 2 % glucose were inoculated with a positive transformant and grown overnight at 37 °C with shaking at 200 r.p.m. To confirm successful transformation of the recombinant pET SUMO vector into *E. coli* BL21 (DE3), 10 µl of the transformation reaction was spread on a prewarmed LB agar plate containing 50 µg ml^−1^ kanamycin and incubated overnight at 37 °C. A single colony was used to set up colony PCR as described previously.

### Overexpression of TccZ from *E. coli* BL21 (DE3)

The expression of TccZ protein from transformed One Shot *E. coli* BL21 (DE3) was performed as follows. Ten microlitres of LB broth supplemented with 50 µg ml^−1^ kanamycin and 2 % glucose were inoculated with either 500 µl of BL21(DE3)/pET SUMO-*tccZ* or 500 µl of BL21(DE3)/pET SUMO-CAT and grown overnight in a shaking incubator (200 r.p.m.) at 37 °C. Fifty microlitres of LB broth containing 50 µg ml^−1^ kanamycin and 2 % glucose were inoculated with 2.5 ml overnight culture of BL21(DE3)/pET SUMO-*tccZ* and BL21(DE3)/pET SUMO-CAT, followed by incubation at 200 r.p.m. and 37 °C until an OD_600_ of 0.5 was reached for each sample. The culture was split: 25 ml as induced (I) and 25 ml as uninduced (UI). One of the 25 µl cultures was induced with a final concentration of 1 mM IPTG and a 1 ml sample was taken from both the uninduced and induced cultures (these samples served as time zero samples). One millilitre samples were collected hourly for 5 and 24 h post-induction. Additionally, the protein expression was repeated as described here with the concentration of IPTG varied at 0.5 mM and 1.5 mM to determine if expression of TccZ would improve.

The samples were spun down at 16 000 ***g*** for 20 min at 4 °C and resuspended in 150 µl room temperature BugBuster reagent and 25 µl of lysozyme. The supernatant (S fraction) was transferred to a clean tube. Protein concentrations were quantified using the Bradford assay [21] and each fraction was separated and assessed on a normalized TGX Stain-Free FastCast acrylamide gel (Bio-Rad) at 90 V for 45 min. Following protein visualization on a Gel Doc EZ imaging system (Bio-Rad, USA), the gels were further processed for Coomassie staining using the rapid Fairbanks staining method [22]. Briefly, the gel was placed in solution A (25 % isopropanol, 10 % acetic acid and 0.05 % Coomassie red) and heated in a microwave until just before boiling, followed by shaking for 5 min. The gel was transferred from solution A into solution B (10 % isopropanol, 10 % acetic acid, 0.0005 % Coomassie red) and then into solution C (10 % acetic acid, 0.002 % Coomassie red) with the heating and shaking steps repeated between transfers. Finally, the gel was rinsed in solution D (10 % acetic acid).

### Results and discussion

### Transformation of One Shot Mach-1-T1 *E. coli* with codon-optimized *tccZ*

**Fig. 1.**
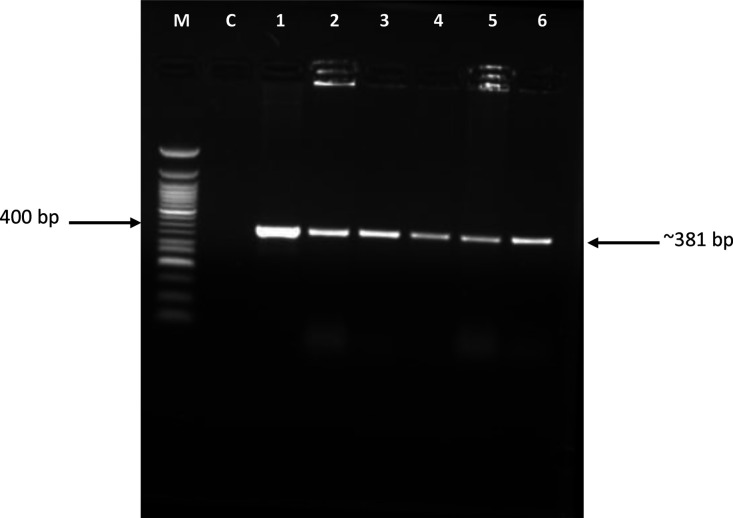
Colony PCR for confirmation of One Shot Mach-1-T1 *E. coli* transformation with the *tccZ* gene. Lane M, 50 bp DNA ladder (New England Biolabs); lane C, no template control. Lanes 1–6, *tccZ*.

The PCR amplicons from individual colonies of the same transformation reaction (Fig. 1, lanes 1–6) appear as single bands of 381 base pairs (bp), which is the expected size of the *tccZ* gene. Following successful transformation albeit at a low transformation efficiency of 4.05×10^4^ c.f.u. µg^−1^, the recombinant cells were named One Shot Mach1-T1/pET SUMO-*tccZ*. These cells were used to maintain the recombinant plasmid.

### Transformation of One Shot *E. coli* BL21 (DE3) with xodon-optimized *tccZ*

For expression of TccZ protein, One Shot *E. coli* BL21 (DE3) cells were used. This *E. coli* strain harbours the lambda DE3 lysogen that carries the gene that encodes T7 RNA polymerase, which is inducible by IPTG, making this strain suitable for protein expression studies [23].

**Fig. 2.**
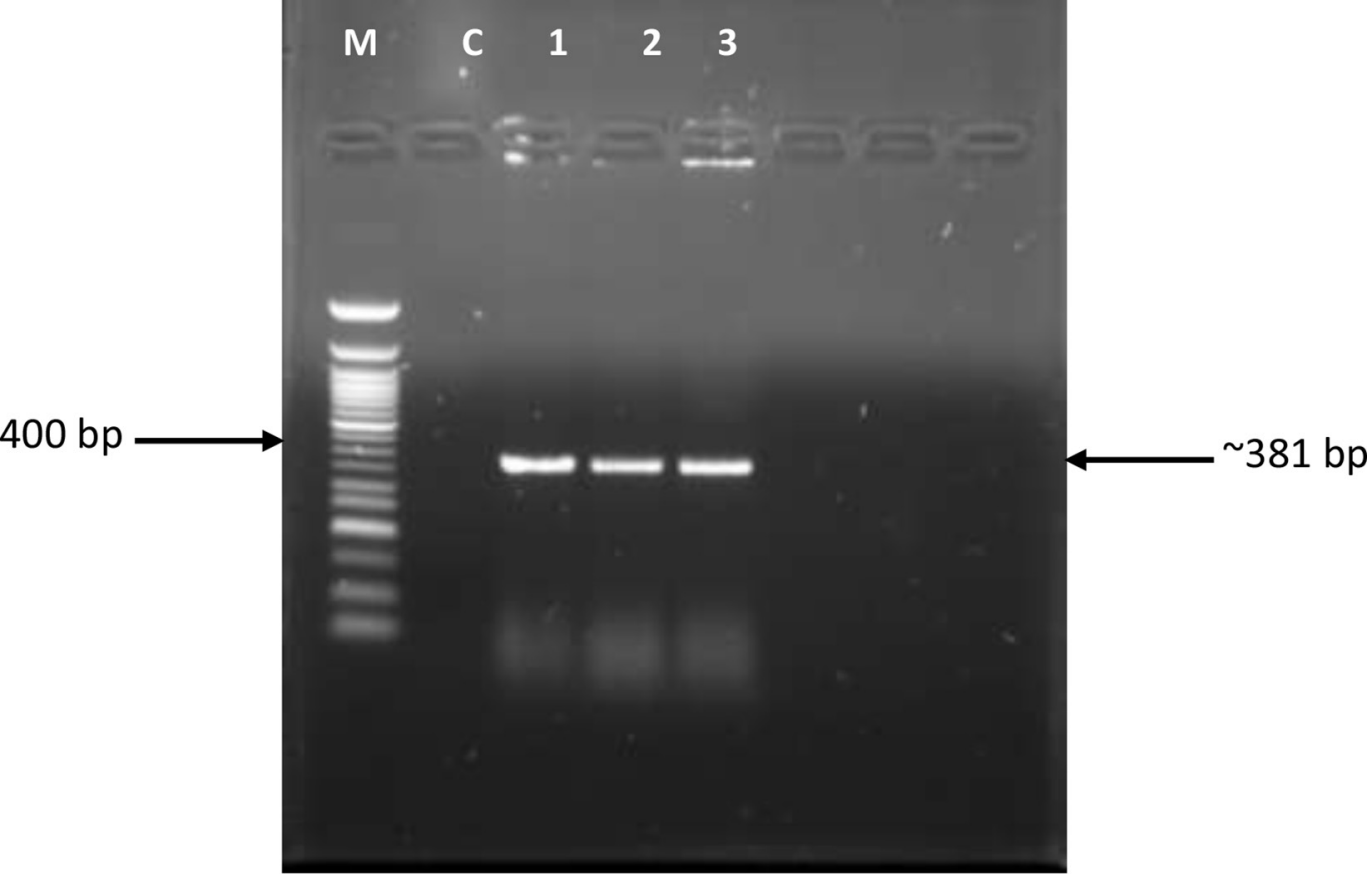
Colony PCR for confirmation of *E. coli* BL21 (DE3) transformation with *tccZ* gene. Lane M, 50 bp DNA ladder (New England Biolabs); lane C, control; lanes 1–3=*tccZ* gene.

The results of colony PCR presented in Fig. 2 indicated the presence of the *tccZ* gene in *E. coli* BL21 (DE3), thereby confirming successful uptake of the recombinant plasmid. Lanes 1, 2 and 3 represent *tccZ* amplicons from individual colonies of the same transformation reaction. The recombinant cells were thereafter referred to as One Shot *E. coli* BL21 (DE3)/pET SUMO-*tccZ* and used for protein expression studies.

### Overexpression of *TccZ* from *E. coli* BL21 (DE3)

The use of insecticidal protein toxins such as vegetative insecticidal proteins (Vip) and crystal proteins (Cry) are at the forefront for the management of detrimental pests of food and other industrial crops. Tc proteins expressed by symbiotic bacteria found in entomopathogenic nematodes [8, 9] are a promising alternative where resistance by insects has emerged, especially in *Bt* transgenic plants. Herein, we transformed *E. coli* with a codon-optimized *tccZ* gene from *P. ananatis* strain MHSD5 with the aim of overexpressing the insecticidal protein toxin and to demonstrate its potential as a biopesticide. The gene was introduced into *E. coli* using a pET SUMO vector (Invitrogen) in which the SUMO protein is known to increase the expression of recombinant proteins as well as enhance their solubility [24, 25].

An initial study was conducted to establish the optimal time required for TccZ expression after induction with 1 mM IPTG. Samples were collected from both induced and uninduced cultures hourly for 5 h and at 24 h post-induction. Total cellular protein was assessed on normalized Stain-Free as well as Coomassie-stained gels. Stain-Free technology has a limit of detection of 0.2–5 ng protein, while Coomassie staining can detect from 6 ng of protein [26]. Based on the length of the *tccZ* gene (381 base pairs), the molecular weight of the TccZ protein was calculated to be 13.97 kDa. The N-terminal peptide containing the poly-histidine tag (His-tag) and SUMO fusion protein increases the size of expressed protein by approximately 13 kDa [23], therefore a fusion TccZ protein of approximately 26.97 kDa was expected.

**Fig. 3.**
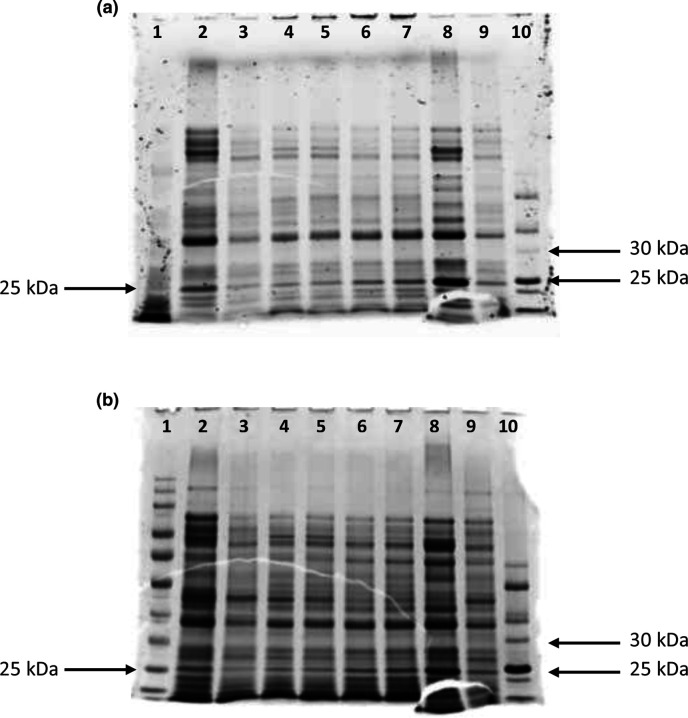
**(a**). Stain-Free and (b) Coomassie-stained normalized SDS-PAGE gel of total protein from One Shot *E. coli* BL21 (DE3)/pET SUMO-*tccZ* induced with 1 mM IPTG and collected at 0, 2, 4 and 24 h. The same samples were separated on both the Stain-Free and Coomassie-stained gels. Lane 1, Colour Pre-stained Protein standard (10–250 kDa), lane 2, T_0UIp_; lane 3, T_0Ip_; lane 4, T_2UIp_; lane 5, T_2Ip_; lane 6, T_4UIp_; lane7, T_4Ip_; lane 8, T_24UIp_; lane 9,T_24Ip_; lane 10, Unstained Protein standard. (10–250 kDa.) I_p_, induced pellet and UI_p_, uninduced pellet.

From Fig. 3, no clear expression of TccZ (expected between 25–30 kDa of the prestained protein standard) was observed as both the uninduced and induced cultures appeared similar in terms of protein distribution. This could have been as a result of basal expression in the uninduced culture despite the T7*lac* promoter being inducible. However, over time there was no intensification of protein band in the vicinity of 26.97 kDa from the induced culture. Therefore, overexpression of the recombinant TccZ protein appears to be absent.

**Fig. 4.**
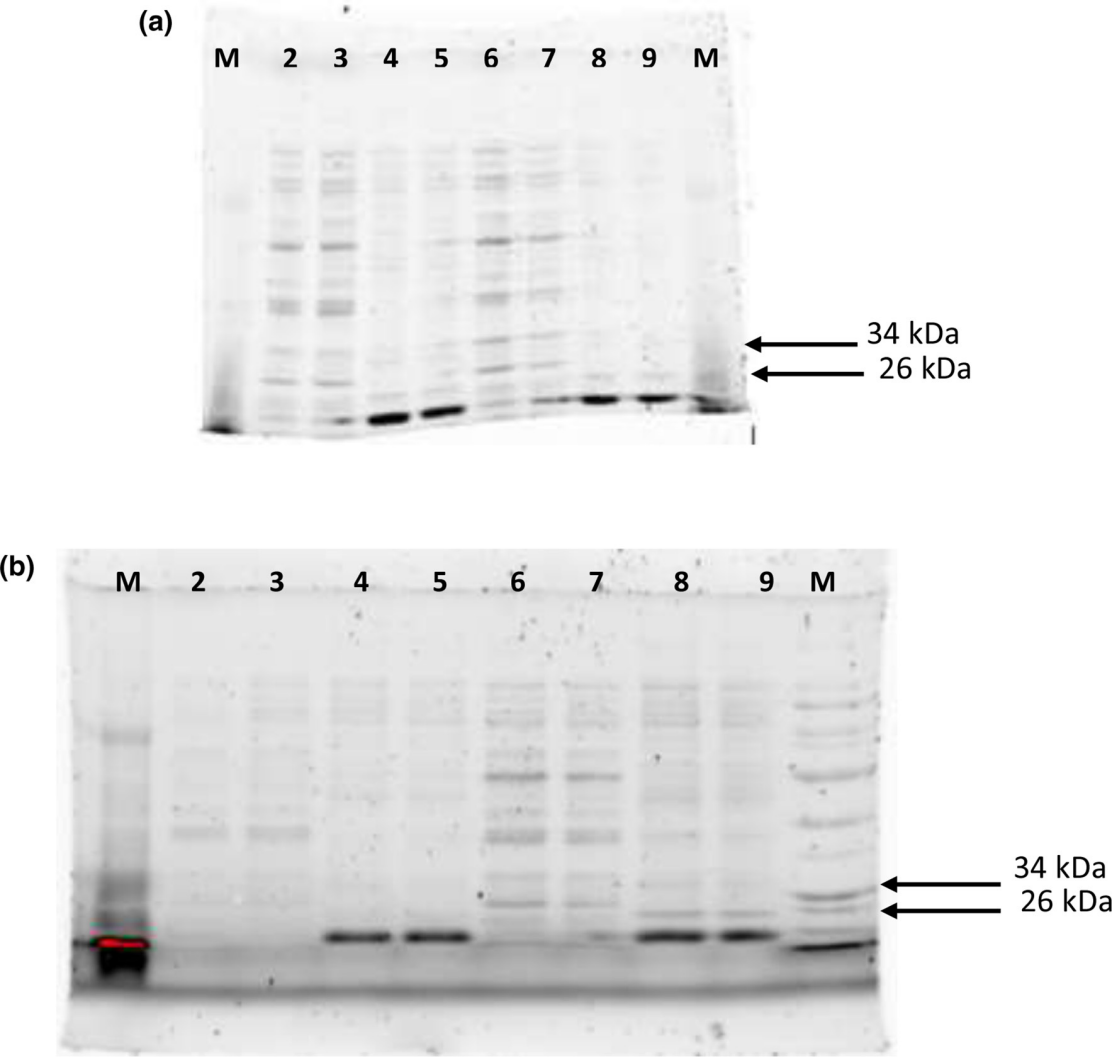
SDS-PAGE analysis of soluble and insoluble protein fractions expressed from One Shot *E. coli* BL21 (DE3)/pET SUMO-*tccZ* induced with 1 mM IPTG. Samples were collected at (**a**) 3 (**t_3_**) and 5 h (**t_5_**) and were separated on both the Stain-Free and Coomassie-stained gels. Lane M, Colour Pre-stained Protein standard (New England Biolabs) (10–250 kDa); lane 2, T_3UIs_; lane 3, T_3Is_; lane 4, T_3UIp_; lane 5, T_3Ip_; lane 6, T_5UIs_; lane7, T_5Is_; lane 8, T_5UIp_; lane 9, T_5Ip._ Samples collected at (**b**) 0 and 24 h are also shown. Lane M, Colour Pre-stained Protein standard (New England Biolabs) (10–250 kDa); lane 2, T_0UIs_; lane 3, T_0Is_; lane 4, T_0UIp_; lane 5, T_0Ip_; lane 6, T_24UIs_; lane7, T_24Is_; lane 8, T_24UIp_; lane 9, T_24Ip._ I_p_, induced pellet; I_s_, induced supernatant; UI_p_, uninduced pellet; UI_s_, uninduced supernatant.

Additional evaluation of the samples for expression of soluble protein also indicated that there was no expression of TccZ in either the soluble or insoluble fractions (Fig. 4). The small ubiquitin-like modifier (SUMO) tag is reported to enhance the solubility of partially insoluble protein, while the BugBuster Extraction Reagent was used to gently disrupt the bacterial cell wall of *E. coli *, enabling the release of the active soluble protein fraction without protein denaturation as well as shielding the protein from oxidation and heat [27].

If *tccZ* was expressed as a soluble protein, an intensified band would have been observed in the lanes representing the inducted supernatant (I_s_, Fig. 4a). However, visible expression of recombinant protein was not evident even 24 h post-induction (Fig. 4b). None of the protein bands within the expected size range could be attributed to expression of TccZ, as similar bands could be seen in the 0 h induced sample. Conversely, if TccZ protein was expressed as an insoluble protein, intensified bands were expected between 10–34 kDa in induced pellet (I_p_, Fig. 4). However, the protein bands observed were similarly present in the 0 h uninduced sample, indicating that this could not be TccZ protein. The *tccZ* gene was cloned in-frame with the sequence of the fusion SUMO tag; this suggests that the expression and solubility of TccZ should have been improved [23, 25]. SUMO acts as a chaperonin similar to ubiquitin and enables stable protein folding to occur in recombinant proteins. Insoluble proteins fused to SUMO have been observed to fold correctly and become soluble [28]. However, this was not the case since the SDS-PAGE results of the present study did not indicate any expression for TccZ protein.

**Fig. 5.**
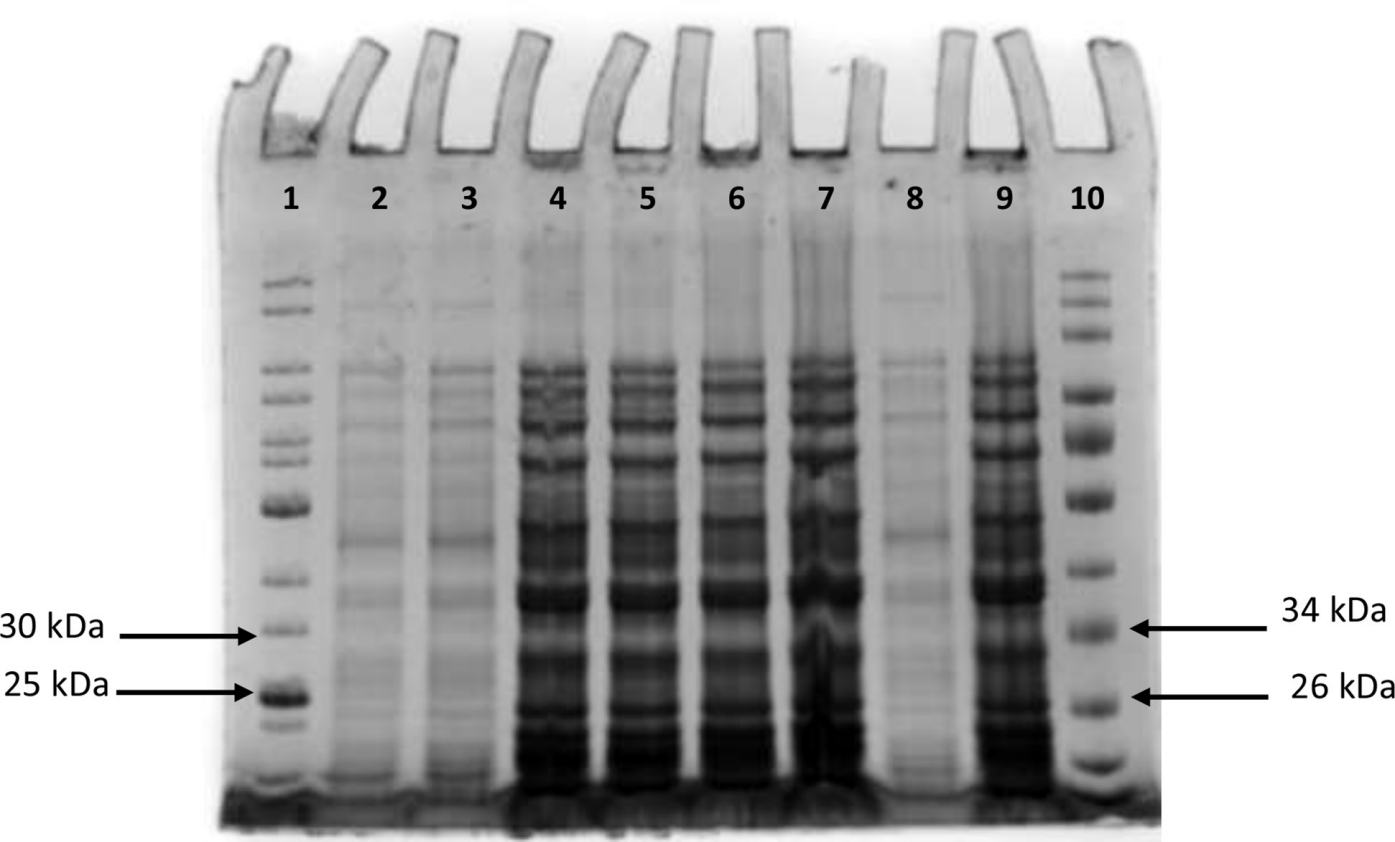
Coomassie-stained SDS-PAGE gel of total cellular protein expressed from One Shot *E. coli* BL21 (DE3)/pET SUMO-*tccZ* induced with 1.5 mM IPTG. Samples were collected at 0, 5 and 24 h from both induced and uninduced cultures. Lane 1, UM [Unstained Protein Standard, 10–250 kDa (New England Biolabs)]; lane 2, UIT_0_; lane 3, IT_0_; lane 4, UIT_5_; lane 5, IT_5_; lane 6, UIT_24_; lane7, IT_24_; lane 8, control IT_0_; lane 9, control UIT_24_; lane 10, M [Colour Pre-stained Protein standard, 10–250 kDa (New England Biolabs)]. I, induced and UI, uninduced.

During the initial expression study, a concentration of 1 mM IPTG was used to induce protein expression from One Shot *E. coli* BL21 (DE3)/pET SUMO-*tccZ* as per the manufacturer’s recommendations. Since no expression was observed in total cellular protein or in the soluble and insoluble fractions, the concentration of IPTG was varied (0.5, 1 and 1.5 mM) to determine if this would optimize the expression of TccZ. Fig. 5 shows a representative Coomassie-stained SDS-PAGE gel of total cellular protein collected from cultures induced with 1.5 mM IPTG. As seen in the figure, none of the samples show TccZ expression.

Low yields of expressed protein as well as failure to express protein of interest is a daunting challenge that is attributed to multiple factors [29, 30]. For example, during heterologous protein expression, a protein coding gene is in an organism that differs from its native host and this is a challenge for proteins that require co-expression with binding protein partners for efficient expression as well as stability [29, 30]. The requirement for binding protein partners is important in this study because the *tccZ* gene belongs to the tcC part of the toxin complex. As such, it is possible that *tccZ* requires other binding partners in the same toxin complex for improved protein expression, and these binding partners might be available in the native host and absent in the expression host. Therefore, it is probable that co-expressing the *tccZ* gene with genes that it interacts with within its native host may improve protein expression. Furthermore, recombinant proteins are perceived by the cell as being unwanted and are subjected to proteolysis, which reduces the number of expressed proteins. This may be alleviated by the use of protective fusions and protein inhibitors [29]. Protein insolubility also limits protein expression and can be improved with co-expression and chaperones as well as the use of stronger and more highly regulated promoters [29, 31].

Some proteins of interest are toxic and may result in the death of the bacterial host cell [32]. In the presence of a toxic target protein, an alternative expression host is necessary. A previous study recommended the use of mutants strains of *E. coli* BL21(DE3), *E. coli* C41(DE3) and C43(DE3) as alternative hosts for the expression of proteins that are poorly expressed in BL21 (DE3) [32]. Comparison of these mutant strains with BL21 (DE3) showed that expression of the same proteins is better in the mutant strains than it is in BL21 (DE3) [32]. It was further demonstrated that when considering plasmid stability, it is preferable to use strain C43(DE3) rather than C41(DE3). It is possible that TccZ could be toxic to the *E. coli* host, but from the normalized SDS-PAGE gel shown in Fig. 3 it is visible that expression of cellular protein increased similarly over time in both induced and uninduced samples. If the expressed protein was toxic, a reduction in bacterial growth and therefore expression would have been evident over the 24 h sampling period. An alternative would be to consider lowering the temperature to 4 or 25 °C at induction or replacing LB medium, which limits cell density, with autoinduction medium, which circumvents the need to monitor cell growth for induction at the correct cell density [33].

On the other hand, a yeast protein expression system may be considered. An example of a yeast host that can be used is *Pichia pastoris*, a preferable alternative expression host due to its strong and tightly regulated promoters, as well as its ability to produce high titres of proteins [32]*.* High-titre protein production is attributed to the ability of *P. pastoris* to grow to high cell densities [32, 34]. It is, however, not known whether expression in a eukaryotic host would subsequently have a negative impact on the activity of the prokaryotic Tc proteins.

This study is not the first study to report undetected protein expression of one of the toxin complex genes. A previous study reported failure to detect expression of one of the genes that belongs to the B subunit of the toxin complex on SDS-PAGE [15]. Another study reported failure to detect C-subunit protein when expressed in the absence of the B-subunit [6]. It is reported that expression of the C-subunit in the absence of B-subunit genes results in lower to no expression of the genes. Although the gene is closely linked to the loci of *tcc* insecticidal toxin, the function of TccZ protein is unknown [10]. To further elucidate the role of TccZ as an insecticidal toxin and any potential for its application as a biopesticide, since TCC components have such potential for toxicity, we attempted to express and purify the protein in *E. coli *. It may be possible that like the C-subunit of the Tc, TccZ, associates with another subunit of the toxin complex to form a binary complex that requires co-expression. This may further explain the failure to express TccZ protein in the present study and warrants further exploration with the co-expression of TCB components in *E. coli *.
